# Rapid homoepitaxial growth of (011) β-Ga_2_O_3_ by HCl-based halide vapor phase epitaxy

**DOI:** 10.1080/14686996.2025.2585551

**Published:** 2025-11-10

**Authors:** Yuichi Oshima, Takayoshi Oshima

**Affiliations:** Research Center for Electronic and Optical Materials, National Institute for Materials Science, Tsukuba, Japan

**Keywords:** β-Ga_2_O_3_, halide vapor phase epitaxy, homoepitaxy

## Abstract

We demonstrated rapid homoepitaxial growth on (011) β-Ga_2_O_3_ substrates using HCl-based halide vapor phase epitaxy (HVPE), in which GaCl was synthesized by reacting metallic Ga with HCl gas, and examined properties of the resulting layer. These were compared with layers grown using Cl_2_-based HVPE, where GaCl was produced from Ga and Cl_2_. The growth rate on (011) substrates, approximately 60% of that on (001), reached ~14 μm/h, which was 5–7 times higher than those previously reported for Cl_2_-based HVPE. Despite this high rate, no polycrystalline grains, sometimes found in Cl_2_-based HVPE, were detected. Atomic force microscopy revealed a surface with root-mean-square roughness of 6.5 nm over a 100 × 100 μm^2^ area. In contrast, Nomarski microscopy revealed the presence of pits (~10 μm in diameter at 3.6 μm thickness) with a density of ~3.7 × 10^3^ cm^−2^, a feature not reported for Cl_2_-based HVPE. Cross-sectional transmission electron microscopy confirmed the absence of crystal defects or inclusions at the pit bottom. X-ray diffraction 2*θ*–*ω* scans and pole figure measurements confirmed that the epitaxial layers were single crystalline, with rocking-curve FWHM values comparable to or smaller than those of the substrate. Secondary ion mass spectrometry revealed a chlorine concentration of 1.7 × 10^15^ cm^−3^, which was significantly lower than 1.1 × 10^16^ cm^−3^ measured in the (001) layers. Thus, while the pit issue requires further investigation, HCl-based HVPE enables the rapid growth of low-chlorine (011) β-Ga_2_O_3_, offering significant potential for cost reduction in high-performance power devices with thick drift layers.

## IMPACT STATEMENT

We achieved rapid homoepitaxial growth of (011) β-Ga_2_O_3_ without structural degradation, while reducing residual chlorine concentration by about one order of magnitude compared to the (001) homoepitaxial layer.

## GRAPHICAL ABSTRACT

**Figure:**
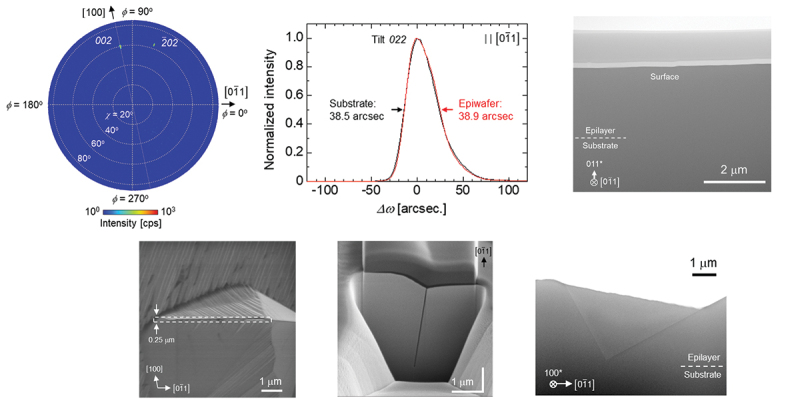

## Introduction

Monoclinic β-Ga_2_O_3_ is an ultra-wide bandgap semiconductor with a bandgap of 4.5–4.9 eV [1,2] and is expected to exhibit a high critical field of ~8 MV/cm [3]. Unlike many other wide bandgap semiconductors, such as GaN and SiC, β-Ga_2_O_3_ can be grown as large bulk single crystals directly from the melt, enabling the use of large-area, high-quality single-crystal substrates for epitaxial growth [4–10]. For these reasons, the realization of high-performance β-Ga_2_O_3_-based power devices with high breakdown voltage and low loss is anticipated, and intensive research and development are underway. Promising device prototypes, such as Schottky barrier diodes [11–13] and metal-oxide-semiconductor field effect transistors [14–17], have been demonstrated.

Realization of high-performance β-Ga_2_O_3_-based power devices requires high-quality epitaxial layers with controlled impurity concentrations. The reported epitaxial growth methods for β-Ga_2_O_3_ include molecular beam epitaxy [18,19], mist chemical vapor deposition [20,21], metal–organic vapor phase epitaxy [22–24], and halide vapor phase epitaxy (HVPE) [25–28]. To grow thick drift layers with low donor concentrations for high-performance β-Ga_2_O_3_-based power devices, epitaxial techniques must provide high growth rates, low background impurity levels, and low cost. HVPE is considered a promising method to satisfy these requirements. Several devices have been fabricated and demonstrated using HVPE technique [11–13,16,17,29–31].

In the HVPE of β-Ga_2_O_3_, GaCl is mainly used as the Ga source material. The GaCl is synthesized by reacting metallic Ga with either Cl_2_ or HCl. In this study, we distinguish between these two processes and refer to them as Cl_2_-based HVPE and HCl-based HVPE, respectively. A key difference is that HCl-based HVPE generates H_2_ as a byproduct during GaCl synthesis, whereas Cl_2_-based HVPE does not. To date, β-Ga_2_O_3_-based power devices have been mainly fabricated and demonstrated using epitaxial layers grown by Cl_2_-based HVPE [16,17,29–31]. This is because Cl_2_-based HVPE provides a hydrogen-free growth atmosphere, suppressing Si contamination originating from reactions between hydrogen-containing species and the quartz reactor tube at high temperatures to below the detection limit of secondary mass spectrometry (SIMS). However, due to the large equilibrium constant of the reaction that produces Ga_2_O_3_, high precursor partial pressures promote parasitic reactions that produce polycrystalline Ga_2_O_3_ particulates. These particulates can impinge on the epitaxial surface and degrade the crystal quality. For example, polycrystalline grains were observed on the surface of (001) β-Ga_2_O_3_ homoepitaxial layers grown at 6 μm/h using Cl_2_-based HVPE [27]. Consequently, commercial epitaxial growth by Cl_2_-based HVPE is typically limited to growth rates of 4–5 μm/h [32].

In HCl-based HVPE, the generation of H_2_ as a byproduct during the GaCl synthesis often results in higher residual Si concentrations than those observed in Cl_2_-based processes. However, as discussed later, relatively low residual Si concentrations of 10^15^–10^16^ cm^−3^ in homoepitaxial β-Ga_2_O_3_ layers can be achieved by the HCl-based process, and we believe this issue is not fundamental, as it can likely be resolved in the future by using Si-free materials for reactor construction. Moreover, in HCl-based HVPE, the presence of H_2_ as an etching gas for Ga_2_O_3_, should suppress particulate formation, even under a relatively high precursor supply, thereby facilitating higher growth rates. This effect can be further enhanced by intentionally adding an appropriate amount of HCl gas to the growth atmosphere [33]. Indeed, as demonstrated later in this paper, HCl-added HCl-based HVPE enables the rapid growth of (001) β-Ga_2_O_3_ homoepitaxial layers with a low Si concentration of 8.6 × 10^15^ cm^−3^ and excellent surface morphology at a high growth rate of 24 μm/h.

To date, demonstrations of high-performance vertical β-Ga_2_O_3_-based power devices have primarily relied on (001) epitaxial layers grown by Cl_2_-based HVPE. The (001) orientation is preferred because large-area substrates can be fabricated using the edge-defined film-fed growth (EFG) method, and twin-free epilayers can be achieved at reasonable growth rates. However, (001) epilayers grown by Cl_2_-based HVPE often exhibit deep pits elongated along the *b*-axis, likely originating from dislocations [28]. The pit length decreases as the off-angle increases [28]. While the pit depth depends on the off-angle toward the *b*-axis, for commercially common (001) substrates with an off-angle of ~0.1°, the pit depth can reach ~40% of the epitaxial layer thickness [32]. Consequently, planarization by chemical mechanical polishing is required before device fabrication, leading to a significant loss of epilayer thickness and added cost. In addition, Cl is easily incorporated as an impurity, which is also a common issue for HCl-based HVPE. The residual Cl concentration is on the order of 10^16^ cm^−3^ under typical growth conditions. Note that the residual Cl concentration further increases at relatively higher growth rates (Figure S1). In typical power devices, high concentrations of residual Cl are detrimental because Cl acts as a shallow donor, limiting the breakdown voltage. Therefore, it is desirable to lower the residual Cl concentration to below 10^16^ cm^−3^.

Recently, the (011) plane has attracted attention as a promising alternative to (001) [32,34–38]. One reason is that, in Cl_2_-based HVPE, dislocation-related pits have been reported to not form on the (011) surface [28]. However, when (011) substrates grown by the vertical Bridgman (VB) method were used, polycrystalline domains formed mainly at the epilayer/substrate interface, with a density of ~80 cm^−2^ under growth conditions identical to those of (001) [36]. Suppressing these defects required the insertion of an intermediate layer grown under different conditions during the initial stage, although the specific growth parameters of this layer have not been disclosed [36]. In contrast, such polycrystalline defects have not been reported for Cl_2_-based HVPE on (011) substrates prepared using the EFG method [28], suggesting that these defects likely originate from characteristic imperfections of VB-grown β-Ga_2_O_3_ substrates rather than from impinging polycrystalline particulates generated by parasitic reactions. Although substrate-induced polycrystalline defects can be suppressed by the intermediate layers, rapid growth exceeding 6 μm/h using Cl_2_-based HVPE can still cause parasitic-reaction-induced particulate impingement, regardless of orientation. An additional advantage of the (011) orientation is its remarkably low residual Cl concentration (~8 × 10^14^ cm^−3^) – approximately one-tenth that of (001) layers grown under the same conditions – at a growth rate of ~2 μm/h [36]. However, reported growth rates of 2–2.8 μm/h for (011) layers correspond to only 50–60% of those reported for (001) layers under identical conditions [28,36]. Considering that ~50 μm-thick drift layers are required for 10 kV-class devices with a carrier concentration of 1 × 10^16^ cm^−3^ [32], increasing the growth rate of (011) epilayers is essential for economic viability.

In this study, we performed rapid homoepitaxial growth on (011) substrates using HCl-based HVPE, which has not been previously reported for (011), and investigated the structural quality, surface morphology, and impurity concentration, of the resulting epilayer. The results were compared with those obtained for (001) epilayers grown using the same growth conditions and with reported (011) growth results via Cl_2_-based HVPE.

## Experimental methods

HCl-based HVPE of (011) homoepitaxial layers was performed on (011) β-Ga_2_O_3_ substrates, which were sliced from bulk β-Ga_2_O_3_ ingots grown via the VB method (Novel Crystal Technology, Inc.). Epitaxial growth was carried out using a custom-built quartz reactor operated under atmospheric pressure at a growth-zone temperature of 1030°C. O_2_ (>99.99995% purity) and GaCl were used as the source materials. GaCl was synthesized upstream in the reactor via the reaction of metallic Ga (>99.99999% purity) with HCl gas (>99.999% purity). The supply partial pressures of O_2_ and GaCl were set to 1.25 kPa and 125 Pa, respectively. In addition, HCl gas was introduced directly into the growth zone at a partial pressure of 188 Pa to suppress parasitic reactions [33,39,40]. N_2_ (dew point <−110 °C) was used as the carrier gas. Unless otherwise noted, the growth time was 15 min. As described in the Results section, the growth rate under these conditions was ~14 μm/h, yielding 3.6 μm-thick homoepitaxial layers after 15 min of growth.

The structural quality of the homoepitaxial layers was examined by X-ray diffraction (XRD) using X’pert PRO MRD (Panalytical, Netherlands), including 2*θ*–*ω* scans, pole figures, and rocking curves (XRCs), and cross-sectional scanning transmission electron microscopy (STEM) using JEM-2100M (JEOL, Japan). Surface morphology was characterized by Nomarski optical microscopy, laser microscopy, field-emission scanning electron microscopy (FE-SEM) using SU8230 (Hitachi, Japan), and atomic force microscopy (AFM) using Jupiter XR (Oxford Instruments, UK). Impurity concentrations were determined by SIMS. Film thickness and growth rate were estimated from the position of the impurity concentration gap between the epilayer and substrate.

## Results and discussion

**Figure 1.**
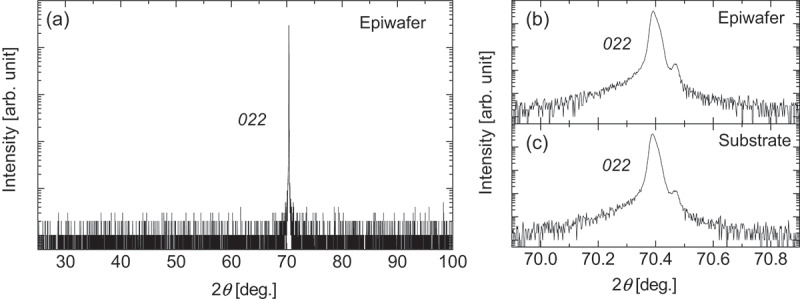
(a) XRD 2*θ*–*ω* scan profile of the (011) homoepitaxial wafer. (b) Magnified view of the region around the *022* peak. (c) XRD 2*θ*–*ω* scan profile of the (011) bare substrate prior to homoepitaxial growth.

Figure 1(a) shows a wide-range XRD 2*θ*–*ω* scan profile of the grown epiwafer in the out-of-plane configuration. No diffraction peaks other than the *022* diffraction of β-Ga_2_O_3_ were observed. Figure 1(b) shows an enlarged view of the *022* peak. A subpeak was detected on the high-angle side of the *022* peak; however, as shown in Figure 1(c), the same subpeak was observed in the substrate prior to epitaxy, indicating that it originated from the substrate. These results confirm that the homoepitaxial layer possesses the out-of-plane orientation expected for a single-crystalline (011) β-Ga_2_O_3_ film.

**Figure 2.**
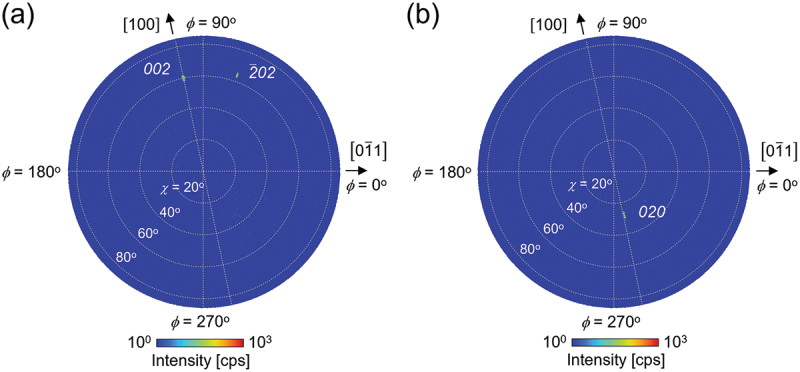
XRD Pole figures of the (011) homoepitaxial wafer measuring (a) *002* and (b) *020* diffractions.

Figure 2(a,b) show XRD pole figures for the *002* and *020* diffractions, respectively. In both cases, peaks were observed only at the positions expected for single-crystalline (011) β-Ga_2_O_3_. Notably, in Figure 2(a), the $\bar{2}02$ diffraction, which has virtually the same 2*θ* value as the *002* diffraction, also appears at the position expected for single-crystalline (011) β-Ga_2_O_3_.

**Figure 3.**
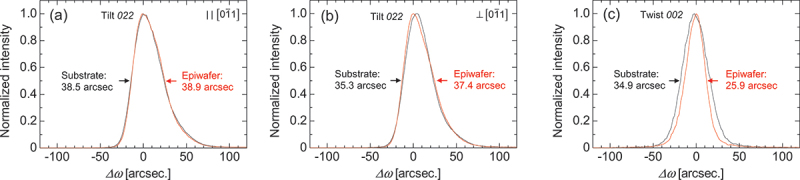
XRCs of the *022* diffraction peak measured in symmetric geometry: (a) along and (b) perpendicular to the $\left[0\overset{ˉ}{1}1\right]$ direction; (c) *002* diffraction measured in skew-symmetric geometry.

Figure 3(a,b) show the XRCs of the *022* diffraction in the symmetric configuration with the rocking direction parallel and perpendicular to $\left[0\overset{ˉ}{1}1\right]$, respectively. The FWHM (tilt angle) serves as an indicator of the mosaicity associated with the inclination of the (011) plane. It should be noted that the *022* XRC peaks exhibited an asymmetric shape with a tail on the right-hand side. The cause of this asymmetric peak shape is not yet clear at this stage. Considering the low-symmetry crystal structure of β-Ga_2_O_3_, it may be possible that the distribution of crystal defects was anisotropic; however, further studies will be required to verify this assumption. Figure 3(c) shows the XRC of the *002* diffraction in the skew-symmetric configuration, where the FWHM (twist angle) represents the mosaicity related to the rotation around the normal of the (011) plane. Since the epilayers were relatively thin, these diffraction peaks included contributions from the substrate. Considering the X-ray penetration depth and absorption coefficient at the CuKα_1_ wavelength, the epilayer contribution to the peak intensity was estimated to be approximately 31% and 80% for the symmetric and skew-symmetric configurations, respectively. Thus, if the crystallinity of the epilayer is substantially degraded compared with that of the substrate, it should be reflected in the FWHM values. The measured FWHM values were comparable to, or even smaller than, those of the substrate, confirming that no degradation of crystalline quality occurred in the epilayer. It is noteworthy that the film thicknesses required to reduce the substrate contribution to the XRC peak intensities of the *022* and *002* diffractions to below 1% are estimated to be more than approximately 46 μm and 10 μm, respectively.

**Figure 4.**
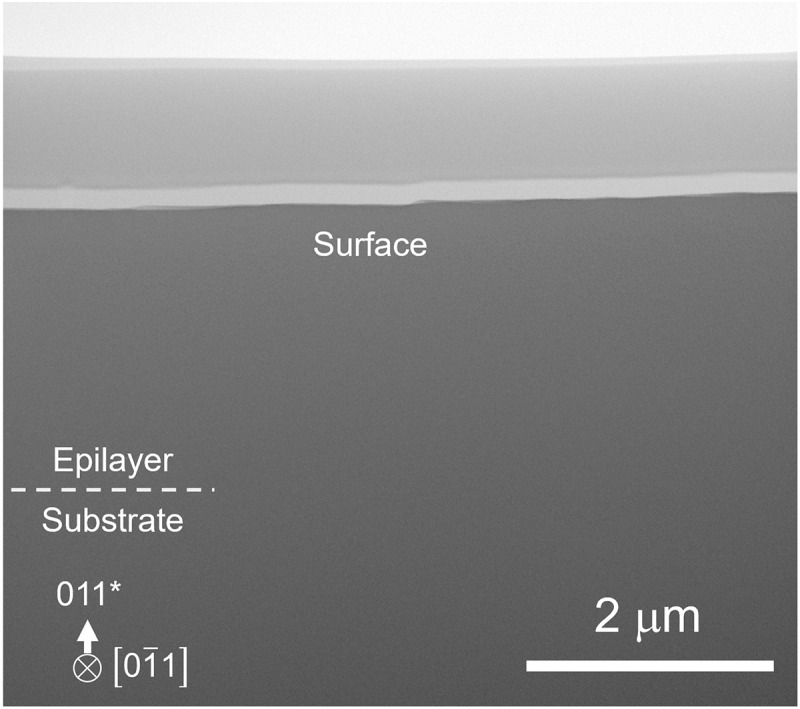
Cross-sectional BF-STEM image showing the epilayer/substrate interface.

Figure 4 shows a cross-sectional bright-field (BF) STEM image (including the epilayer/substrate interface) of an epilayer grown for 5 min in a rectangular window (1 mm × 10 mm) of a SiO_2_ mask formed on a (011) β-Ga_2_O_3_ substrate. The location of the epilayer/substrate interface was determined by horizontally extrapolating the position of the SiO_2_ mask in the sample shown in Figure S2 to the observation area presented in Figure 4. No defects were observed at the epilayer/substrate interface. As shown in Figure S2, the thickness of the (011) epilayer grown in the mask window was significantly larger than that of the epilayer grown without a mask (1.2 μm), particularly near the mask edges. Since no nucleation occurred on the mask, the observed enhancement can be attributed to precursor species that diffused from the mask surface into the window region, rather than being consumed on the mask, thereby contributing to epitaxial growth. The STEM measurement was carried out at a position approximately 30 μm away from the mask edge, where the film thickness had decayed to an approximately constant value. Even at this location, the thickness remained as large as 2.4 μm, corresponding to a growth rate of 29 μm/h. The absence of defect formation at the interface despite this high rate strongly suggests that the (011) orientation has excellent potential for high-quality, high-speed epitaxial growth.

**Figure 5.**
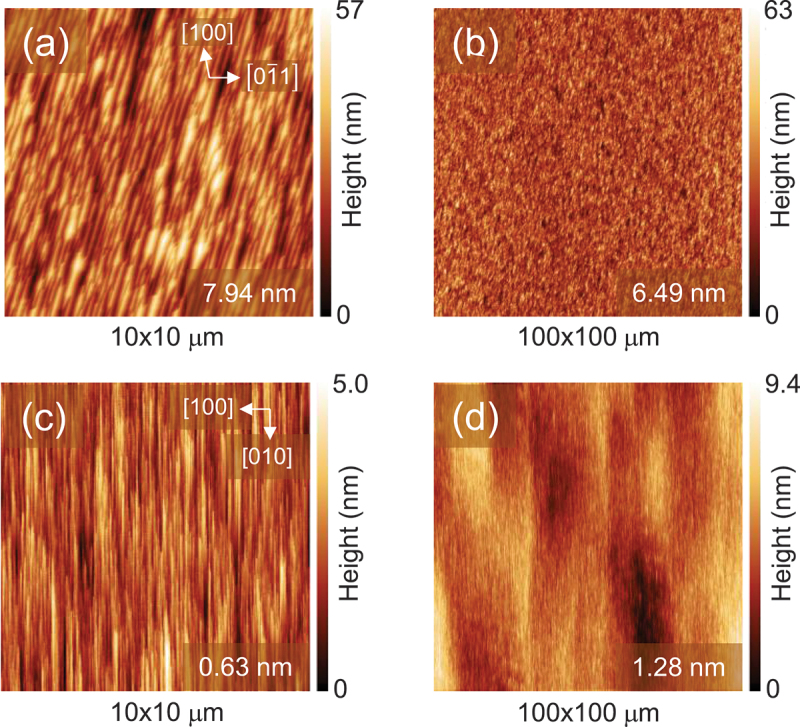
AFM surface images of β-Ga_2_O_3_ homoepitaxial layers. (a) and (b): (011) plane (3.6-μm thick); and (c) and (d): (001) plane (6-μm thick). The rms roughness values are shown in the lower right corner of each image.

Figure 5(a,b) show the AFM images of the (011) epilayer surface. For comparison, the AFM images of the (001) epilayers grown using the same recipe are shown in Figure 5(c,d). The (001) epilayers exhibited a line-like morphology elongated along the *b*-axis, whereas the (011) epilayers displayed line-like features oriented at ~75° from the $\left[0\overset{ˉ}{1}1\right]$ direction, deviating slightly from the $\left[1\overset{ˉ}{1}1\right]$ direction at 71.7°. The root-mean-square (RMS) roughness of the 3.6-μm-thick (011) epilayer over a 100 × 100 μm^2^ area was 6.5 nm, which was significantly larger than that of 6-μm-thick (001) epilayers grown under the same conditions. Notably, the morphology of the (001) epilayers observed for Cl_2_-based HVPE was considerably rougher than those shown in Figure 5(c), most likely due to dislocation-related pits [28]. In contrast, the (011) epilayer surfaces obtained in this study were considerably smoother, except for the pits described later.

**Figure 6.**
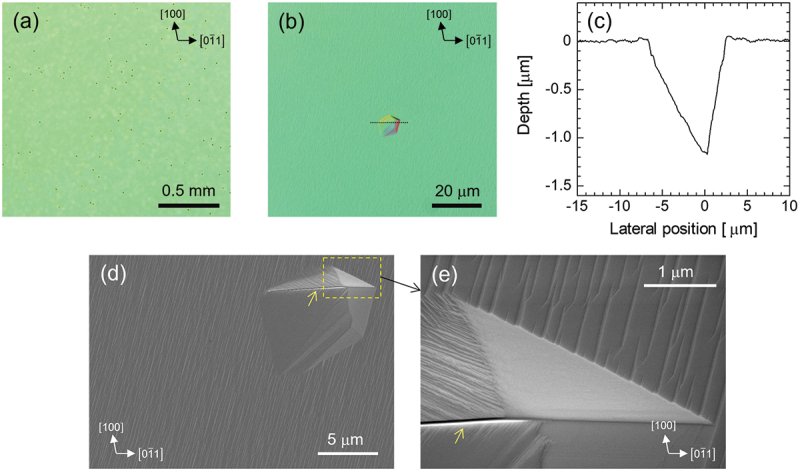
(a) and (b) Nomarski microscopy images of the (011) homoepitaxial wafer. (c) Depth profile of a pit measured along the dotted line in (b) using laser microscopy. (d) and (e) Plan-view SEM images of a pit. Yellow arrows indicate the slit formed along the ridge line of the pit.

Figure 6(a,b) show the Nomarski optical microscopy images of the (011) epilayer surface. Polycrystalline defects, which have been reported in Cl_2_-based HVPE films grown on VB-grown (011) substrates [36], were absent. Contrastingly, numerous pits, unreported in Cl_2_-based HVPE, were observed. The pit density was ~3.7 × 10^3^ cm^−2^, although for substrates from different production lots, it occasionally increased by approximately a factor of three. Figure 6(c) presents the cross-sectional profile of a pit measured by laser microscopy, showing a depth of ~1.2 μm. Since the pit size and depth were relatively uniform, they were likely formed during a specific stage of epitaxial growth, probably near the epilayer/substrate interface. These observations suggest that the pits originated from the features present on the substrate surface. Notably, the fabrication of (011) substrates, from bulk growth via the VB method through subsequent polishing, remains less technologically mature than that of (001) or (010) substrates prepared by the EFG method. Figure 6(d,e) show plan-view SEM images of a representative pit. A distinctly identifiable feature is the presence of a narrow and deep slit, measuring several tens of nanometers in width along the ridge, as indicated using yellow arrows in Figure 6(d,e). Because the slit is extremely narrow and, as described later, not perpendicular to the substrate surface, it does not appear in the depth profile shown in Figure 6(c).

**Figure 7.**
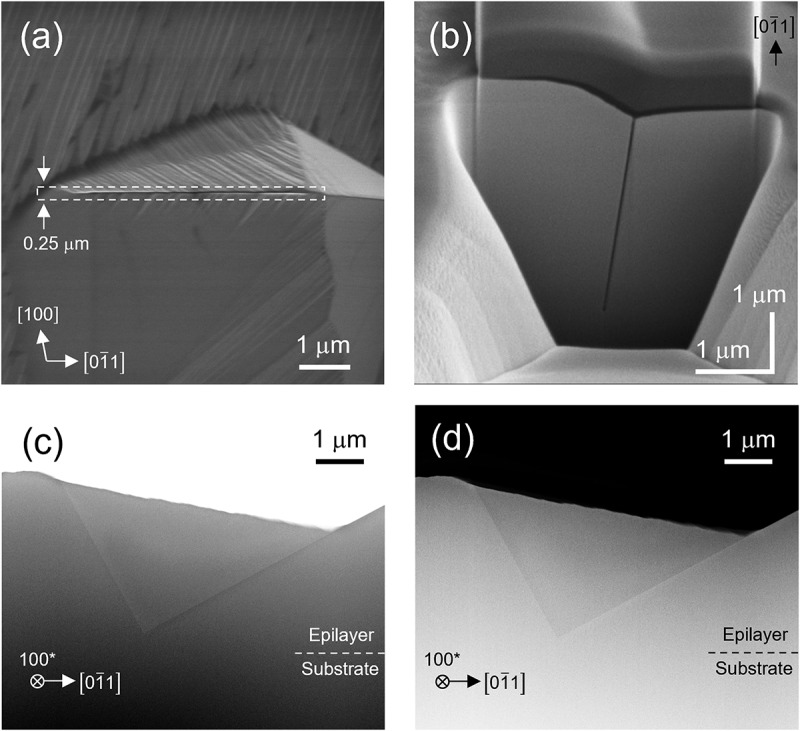
(a) Plan-view sem image of a pit. (b) Sem image of the cross-section of the slit cut perpendicularly to the $\left[0\overset{ˉ}{1}1\right]$ direction (viewed at a tilt angle of 54° from the surface normal). (c) and (d) cross-sectional bf- and HAADF-STEM images of the slit viewed along the [100] direction, respectively. The specimen (thickness = 250 nm) was cut along the *a*-plane, as indicated by the dotted rectangle in (a).

To clarify the cross-sectional geometry and origin of the slit, the cross section was exposed using focused ion beam (FIB) processing and examined by SEM. Figure 7(a) shows a plan-view SEM image of the pit, and Figure 7(b) presents an SEM image of a cross-section perpendicular to the slit, observed at a tilt of 54° from the surface normal. From this image, the inclination angle of the slit with respect to the (011) plane was calculated as 83.1°. Because the angle between the (011) and (100) planes is 83.5°, the slit sidewalls were identified as (100) planes. The (100) plane is the most stable surface of β-Ga_2_O_3_ and exhibits the lowest growth rate [18,41]. In general, surfaces with low growth rates tend to extend more widely. For example, in selective area growth on a *c*-plane β-Ga_2_O_3_ substrate, fins with well-developed (100) side facets can be fabricated by appropriately setting the mask orientation [39]. Based on these considerations, it is likely that once the (100) plane appears for some reason, it expands significantly, leading to the formation of the slit. To investigate the slit geometry along the $\left[0\overset{ˉ}{1}1\right]$ direction, a 250 nm-thick lamella parallel to the slit sidewall (as indicated by the dotted rectangle in Figure 7(a)) was prepared using FIB and examined using cross-sectional STEM. Figure 7(c,d) show the BF-STEM and high-angle annular dark field (HAADF) STEM images, respectively. The epilayer/substrate interface is indicated based on the film thickness estimated from the SIMS depth profile (not shown). Cross-section of the slit exhibited a triangular shape, with its left and right edges inclined by ~61° and ~29°, respectively, in the $\left[0\overset{ˉ}{1}1\right]$ direction. Given that the angles between the $\left[0\overset{ˉ}{1}1\right]$ and $\left[0\overset{ˉ}{1}0\right]$ directions and between the $\left[0\overset{ˉ}{1}1\right]$ and [001] directions are 62.3° and 27.7°, respectively, these edges should be parallel to the *b*- and *c*-axes. The slit apex was located at a depth of ~3.2 μm from the (011) surface, shallower than the epilayer/substrate interface, and no crystal defects or foreign particles were observed in its vicinity. Therefore, crystal defects or impurities can be excluded as the origin of the slits and pits; however, their precise origin remains unclear and requires further investigation.

Table 1 summarizes the SIMS results of the impurity concentrations in the (011) epilayers compared to those of the (001) epilayers grown under the same conditions. Although [Si] was higher in the (011) epilayer than that in the (001) epilayer, both [N] and [Cl] were lower. In particular, as in Cl_2_-based HVPE, [Cl] was strongly suppressed. Thus, if [Si] can be further reduced, residual donor concentrations low enough for realizing 10 kV-class devices should be achievable. Based on the position of the impurity concentration gap at the epilayer/substrate interface in the depth profile (not shown), the film thickness was estimated to be 3.6 μm. This corresponds to a growth rate of ~14 μm/h, which is approximately 60% of that for (001) under identical conditions. This reduction in growth rate on (011), including its ratio to (001), is consistent with previous reports for Cl_2_-based HVPE [28,36]. Nevertheless, high-speed growth, approximately 5–7 times faster than that reported for Cl_2_-based HVPE, was achieved without generating polycrystalline particulates, which was highly beneficial for reducing the cost of thick drift layer fabrication. Our investigation further revealed that in (001) epilayers, [Cl] increases slightly superlinearly with the growth rate (Figure S1). The growth rate dependence of [Cl] in (011) remains to be clarified. However, if the growth rate dependence is assumed to follow the same trend as in (001), then, considering that our growth rate is approximately seven times higher than that reported by Ema et al. [36], the [Cl] in HCl-based HVPE should exceed 5.6 × 10^15^ cm^−3^. In practice, however, the achieved [Cl] was much lower, suggesting that the mechanism of Cl incorporation is dependent on crystal orientation.

**Table 1.** Results of the SIMs measurements.

Element	(011)	(001)
[H] (cm^−3^)	<5 × 10^16^	<5× 10^16^
[N] (cm^−3^)	<1 × 10^16^	5.5 × 10^16^
[Si] (cm^−3^)	2.0 × 10^16^	8.6 × 10^15^
[Cl] (cm^−3^)	1.7 × 10^15^	1.1 × 10^16^

## Conclusion

Homoepitaxial layers were successfully grown on (011) β-Ga_2_O_3_ substrates via HCl-based HVPE. The 3.6 μm-thick (011) epilayer exhibited an RMS roughness of 6.5 nm over a 100 × 100 μm^2^ area, indicating a smooth surface morphology. Unlike Cl_2_-based HVPE on VB-grown (011) substrates, no polycrystalline defects were observed; however, pits with narrow slits bounded by (100) planes appeared at a density of ~3.7 × 10^3^ cm^−2^. Cross-sectional STEM confirmed that no crystal defects or foreign particles were present at the deepest part of the slits, eliminating them as the cause. Despite the pit issue, XRD 2*θ*–*ω* scans and pole figure measurements verified that the homoepitaxial layers were single crystalline without misoriented domains. FWHM values determined from the XRCs showed no significant increase compared with those of the substrates, confirming that homoepitaxy preserved structural quality. Cross-sectional STEM further revealed that no defects formed at the epilayer/substrate interface. The growth rate of the (011) epilayers was ~14 μm/h, approximately 60% of that for (001) epilayers under the same conditions, yet still considerably higher than previously reported values for Cl_2_-based HVPE. Moreover, the residual [Cl] was reduced to 1.7 × 10^15^ cm^−3^, an order of magnitude lower than the 1.1 × 10^16^ cm^−3^ measured for (001) epilayers grown under identical conditions. In summary, although the pit issue remains to be addressed, HCl-based HVPE enables high-speed growth of high-quality (011) homoepitaxial layers with low [Cl], which we believe will greatly contribute to the realization of the cost-effective fabrication of high-performance β-Ga_2_O_3_ power devices.

## Supplementary Material

Supplemental Material
