# Supplementary material for: Rapid homoepitaxial growth of (011) β-Ga2O3 by HCl-based halide vapor phase epitaxy
Source: Sci Technol Adv Mater. 2025 Nov 10;26(1):2585551. doi: 10.1080/14686996.2025.2585551 (PMC12667350; doi:10.1080/14686996.2025.2585551)
Supplement: Supplemental Material [file TSTA_A_2585551_SM9571.docx]

# Figure S1


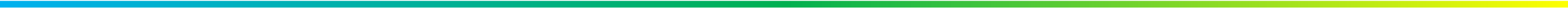


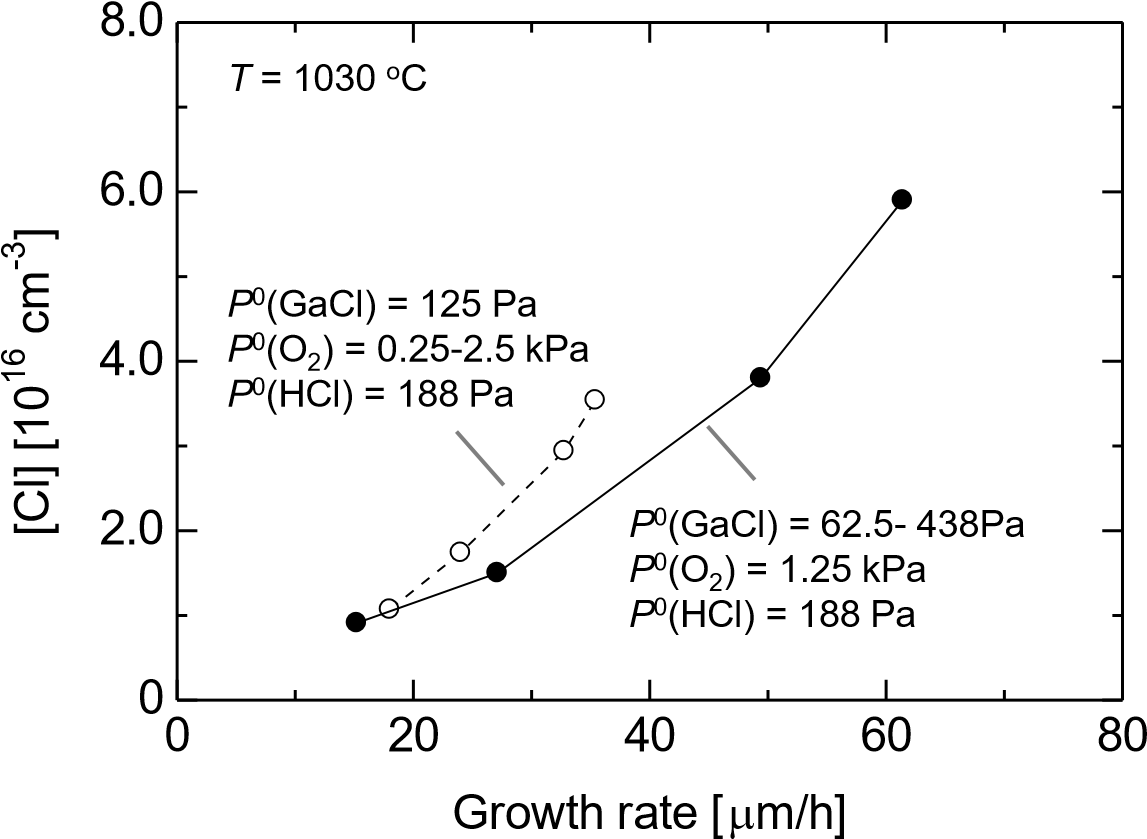


**Figure S1.** Cl concentration in (001) β-Ga_2_O_3_ homoepitaxial layers as a function of growth rate at a substrate temperature of 1030 ^o^C. *P*^0^(GaCl), *P*^0^(O_2_), and *P*^0^(HCl) denote the partial pressures of the precursors and additional HCl supply.

# Figure S2


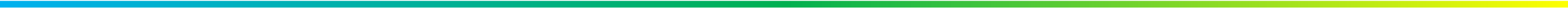

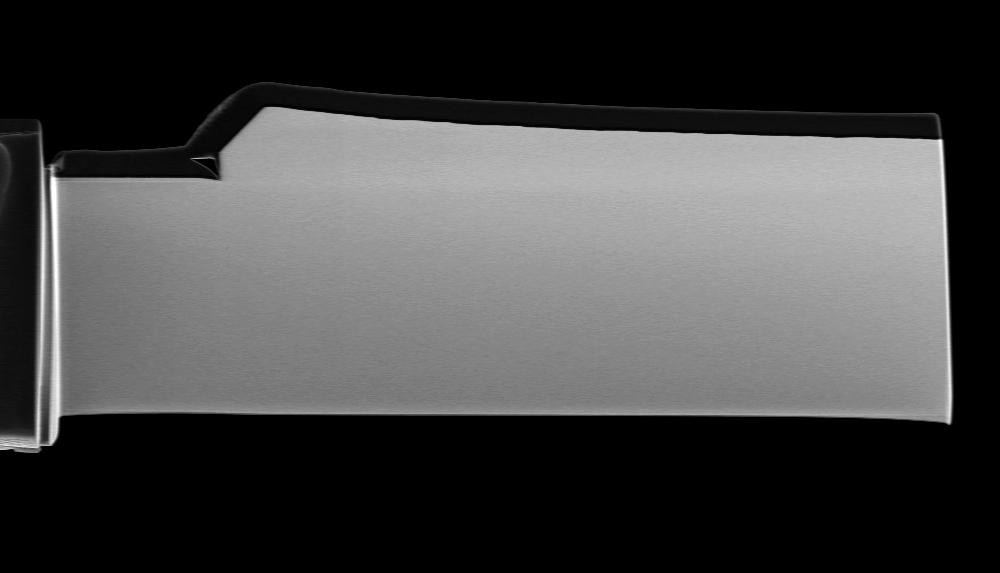


10



m

SiO

2

mask

Substrate

Epilayer

0

ത

1

1

011*

Figure 4

**Figure S2.** Cross-sectional SEM image of the specimen prepared for STEM observation corresponding to **Figure 4**.
